# Capability of Ophthalmology Residents to Detect Glaucoma Using High-Dynamic-Range Concept versus Color Optic Disc Photography

**DOI:** 10.1155/2017/8209270

**Published:** 2017-06-27

**Authors:** Mantapond Ittarat, Rath Itthipanichpong, Anita Manassakorn, Visanee Tantisevi, Sunee Chansangpetch, Prin Rojanapongpun

**Affiliations:** Department of Ophthalmology, Chulalongkorn University and King Chulalongkorn Memorial Hospital, 1873 Rama IV Rd., Pathumwan, Bangkok 10330, Thailand

## Abstract

**Background:**

Assessment of color disc photograph (C-DP) is affected by image quality, which decreases the ability to detect glaucoma. High-dynamic-range (HDR) imaging provides a greater range of luminosity. Therefore, the objective of this study was to evaluate the capability of ophthalmology residents to detect glaucoma using HDR-concept disc photography (HDR-DP) compared to C-DP.

**Design:**

Cross-sectional study.

**Methods:**

Twenty subjects were classified by 3 glaucoma specialists as either glaucoma, glaucoma suspect, or control. All C-DPs were converted to HDR-DPs and randomly presented and assessed by 10 first-year ophthalmology residents. Sensitivity and specificity of glaucoma detection were compared.

**Results:**

The mean ± SD of averaged retinal nerve fiber layer (RNFL) thickness was 74.0 ± 6.1 *μ*m, 100.2 ± 9.6 *μ*m, and 105.8 ± 17.2 *μ*m for glaucoma, glaucoma suspect, and controls, respectively. The diagnostic sensitivity of HDR-DP was higher than that of C-DP (87% versus 68%, mean difference: 19.0, 95% CI: 4.91 to 33.1; *p* = 0.014). Regarding diagnostic specificity, HDR-DP and C-DP yielded 46% and 75% (mean difference: 29.0, 95% CI: 13.4 to 44.6; *p* = 0.002).

**Conclusions:**

HDR-DP statistically increased diagnostic sensitivity but not specificity. HDR-DP may be a screening tool for nonexpert ophthalmologists.

## 1. Introduction

Glaucoma is a chronic progressive optic neuropathy that is characterized by loss of retinal nerve tissue and field of vision. Due to the asymptomatic nature of the disease, most patients are not aware that they have glaucoma until the late stage of the disease. Currently, more than 8.4 million people worldwide are bilaterally blind from glaucoma [1]. It has been estimated that worldwide prevalence of glaucoma will rise to 111.8 million by 2040, with a high proportion in Asia and Africa [2, 3]. However, the actual prevalence of glaucoma may be higher than this estimation, because more than half of glaucoma patients are under diagnosed [4–6]. Early diagnosis and treatment is the key to preventing blindness from glaucoma. One of the highest sensitivity methods for monitoring early glaucomatous change is detection of retinal nerve fiber layer (RNFL) defect [7–9]. Color optic disc photography (C-DP) is a standard tool for RNFL evaluation due to its convenient, low-cost, and noninvasive technique. However, the ability to detect glaucoma, especially by clinicians lacking expertise in glaucoma, has been hindered by the poor quality of C-DP images (e.g., light exposure, poor contrast, and color tone), which are frequently found in media opacity and tigroid fundus cases [10]. High-dynamic-range (HDR) imaging is a computerized technique that was developed to produce a greater dynamic range of luminosity, compensate for loss of detail by adapting different exposure levels, and integrate those exposure levels to reproduce a new image with broader tonal range [11, 12]. Accordingly, the aim of this study was to evaluate the capability of ophthalmology residents to detect glaucoma and peripapillary RNFL defect using HDR optic disc photography (HDR-DP), as compared to detection using C-DP.

## 2. Materials and Methods

**Figure 1:**
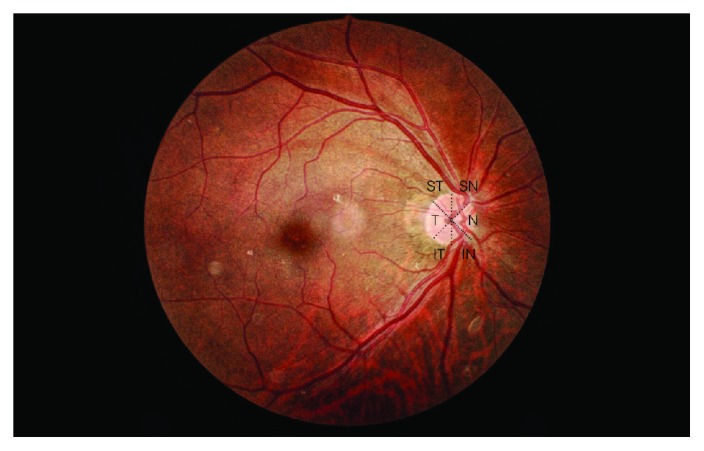
Locations of peripapillary retinal nerve fiber layer. N: nasal; SN: superonasal; ST: superotemporal; T: temporal; IT: inferotemporal; IN: inferonasal.

This cross-sectional study was conducted at the Department of Ophthalmology, King Chulalongkorn Memorial Hospital (Bangkok, Thailand) and was approved by the Institutional Review Board of the Faculty of Medicine, Chulalongkorn University. This study was conducted in accordance with the Declaration of Helsinki and all of its subsequent amendments. There were 3 groups of participants in this study, including glaucoma patients, glaucoma suspect patients, and healthy volunteers. Color optic disc photographs were taken from the eyes of participants in all 3 subgroups. Evaluators consisted of 10 first-year ophthalmology residents. After receiving permission to access and retrieve images, color disc photographs of glaucoma and glaucoma suspect patients were recruited from our hospital's imaging database. Written informed consent was obtained from healthy volunteers prior to their participation in the study. All subjects were more than 18 years old and had a spherical refractive error between −6 diopters and +6 diopters and astigmatism of less than 3 diopters. Exclusion criteria were history of intraocular trauma, retinal disease, neurological disease, and uncooperative subject. All glaucoma and glaucoma suspect subjects had undergone C-DP (KOWA Company Ltd., Nagoya, Aichi, Japan), optical coherence tomography (OCT) (Cirrus-HD OCT; Carl Zeiss Meditec, Dublin, CA, USA), and standard automated perimetry (SAP) (Carl Zeiss Meditec, Dublin, CA, USA) within 6 months prior to the start of the study. All C-DPs from the glaucoma and glaucoma suspect groups were performed in dilated condition by a single experienced photographer at dimensions and settings of 1600 × 1216 pixels and RGB color space in JPEG format. Only qualified images were recruited. Glaucoma patient was defined as vertical cup-to-disc ratio greater than 97.5th percentile of normal population with presence of RNFL defect in OCT or disc photograph that correlated with visual field defect in SAP. Presence of vertical cup-to-disc ratio between 97.5th and 99.5th percentiles of normal population without RNFL defect or functional visual field loss and IOP of less than 21 mmHg were classified as glaucoma suspect subjects. Healthy volunteers who participated in this study had visual field tests and images taken following the same process by the same single photographer. Healthy participants had a vertical cup-to-disc ratio of less than 97.5th percentile with a normal reliable SAP result. Nonqualifying images, OCT, and visual fields included the following: (1) images with poor visualization of retinal blood vessels; (2) OCT scans with incorrect ONH detection, off-centered ONH, motion artifacts, wrong segmentation, or signal strength less than 6; and (3) unreliable SAP with fixation loss, false-positive or false-negative responses greater than 20%. Images from 10 glaucoma patients, 5 glaucoma suspect patients, and 5 healthy volunteers were taken, recorded, and included in the study. A total of 20 C-DPs from all subjects were recruited from April to October 2014. Three glaucoma specialists (VT, AM, and SC) evaluated C-DP, OCT, and SAP to confirm the diagnosis and to identify the number and affected areas of peripapillary RNFL defect in six quadrants (nasal, superonasal, superotemporal, temporal, inferotemporal, and inferonasal) (Figure 1). The determinations reached by the 3 specialists were marked as references for further evaluation by the ophthalmology residents.

**Figure 2:**
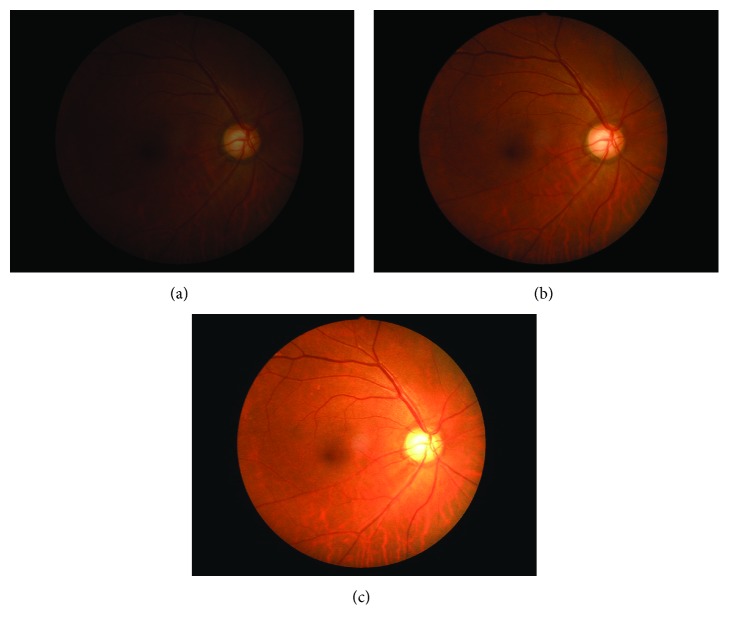
High-dynamic-range image processing of color disc photography with three different exposure levels. (a) Underexposed image; (b) normally exposed image; (c) overexposed image.

All recruited C-DPs were then processed into HDR photos by adjusting the light exposure as overexposed, normally exposed, or underexposed (Figure 2).

**Figure 3:**
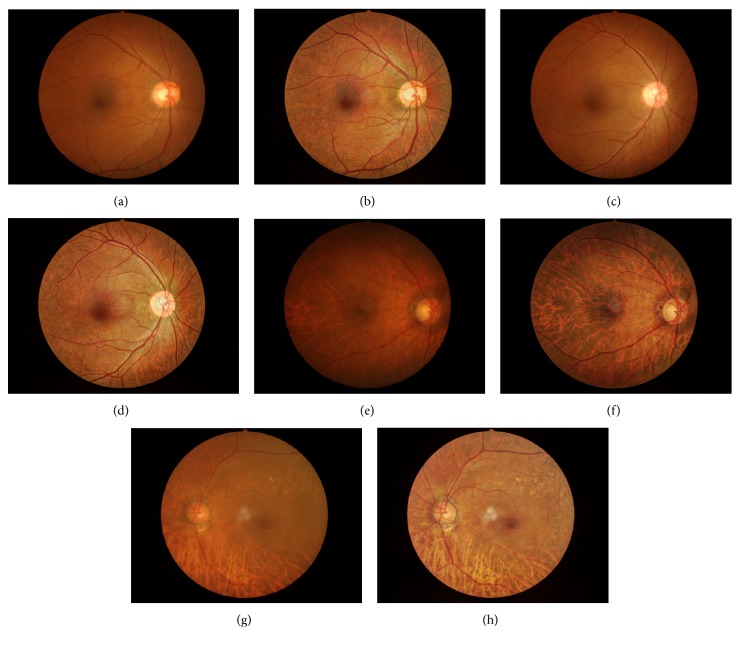
Examples of color and high-dynamic-range disc photographs of 2 normal controls (a, b and c, d) and 2 glaucoma patients (e, f and g, h). Left column (a, c, e, and g) color disc photograph and right column (b, d, f, and h) high-dynamic-range concept disc photograph.

Image editing was performed using preview program for Mac OS X version 8.0. Three different exposure images were then combined using the following settings: strength −4.4, brightness 0.4, local contrast 8.4, white Clip 1.0, black Clip 0, midtone 0, and color saturation −1.5 (Figure 3).

After that, the ten first-year ophthalmology residents who volunteered to join the study provided written informed consent to participate as evaluators. All evaluators were trained in how to diagnose glaucoma and identify RNFL defect. Evaluators were blinded to patient clinical information and diagnosis. The ophthalmology resident evaluators independently assessed a set of 40 randomly sequenced disc photographs consisting of 20 C-DPs and 20 HDR-DPs. Images were projected onto a projector screen at a resolution of 1280 × 800 pixels with 32-bit color in a dark room, and all of the evaluators assessed the image within a period of 30 seconds. The residents were asked to answer 2 questions within 30 seconds, as follows: determine whether the presenting image is glaucoma or not and identify the number and location of RNFL defect.

## 3. Statistical Analysis

All statistical analyses were performed using SPSS version 17.0 (SPSS Inc., Chicago, IL, USA). Paired *t*-test was used to test statistical differences of sensitivity and specificity between C-DP and HDR-DP, as evaluated by the residents when using the glaucoma specialists' references. The primary outcomes were mean difference ± SD of sensitivity and specificity of the test. *p* values less than 0.05 were considered statistically significant.

## 4. Results

Demographic and clinical data of study subjects are shown in Table 1.

**Figure 4:**
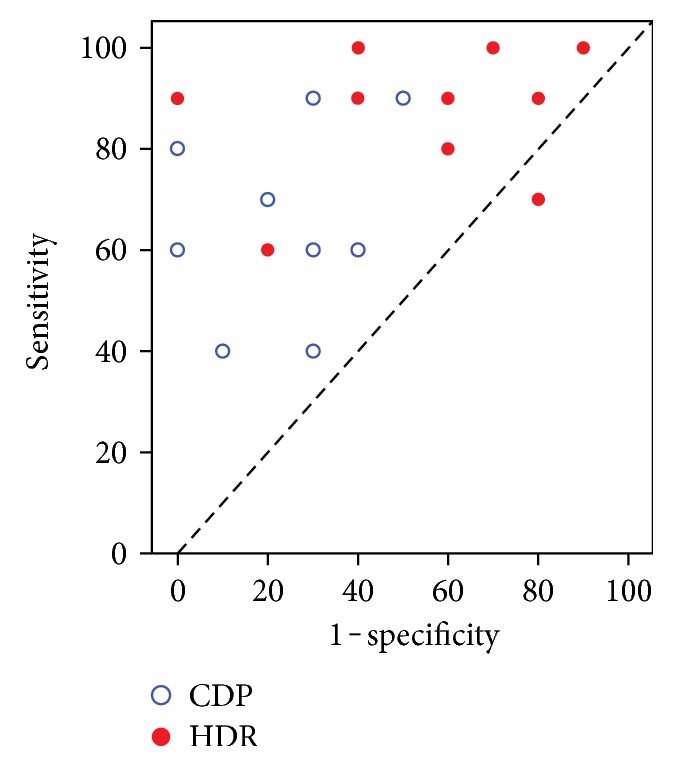
Scatter plot of distribution of sensitivity and specificity in glaucoma diagnosis between C-DP and HDR-DP.

All subjects had best-corrected visual acuity better than 20/30. Distribution of sensitivity and specificity of glaucoma diagnosis using C-DP and HDR-DP by the 10 ophthalmology residents is shown in Figure 4. The scatter plot graph shows high level of distribution for sensitivity in HDR-DP, compared to medium level of distribution for specificity.

The average sensitivity of glaucoma detection in HDR-DP was better than that in C-DP (87% ± 13.4% and 68% ± 19.3%, resp.). The mean difference of sensitivity was 19.0 ± 19.7 (95% CI: 4.91 to 33.1). The average specificity of glaucoma detection in HDR-DP and C-DP was 46% ± 28.8% and 75% ± 17.2%, respectively. The mean difference in specificity was 29.0 ± 21.8 (95% CI: 13.4 to 44.6) (Table 2).

The average sensitivity of RNFL defect detection between C-DP and HDR-DP in each quadrant of the glaucoma group was analyzed, but no statistical significant difference was observed (Table 3). Interobserver agreement in our study was 0.33 (95% CI: 0.21 to 0.45) and 0.43 (95% CI: 0.31 to 0.56) for HDR-DP and C-DP, respectively.

## 5. Discussion

In this study, we applied the HDR technique and evaluated diagnostic accuracy compared with original C-DP in nonexperienced trainees. We found that HDR-DP had higher sensitivity for detecting glaucoma than C-DP, but specificity for detection of glaucoma using HDR-DP was lower than that using C-DP. RNFL defect detection was similar between the two techniques.

Color disc photograph is the backbone diagnostic method for glaucoma diagnosis and is widely used in clinical practice. To improve the visualization of optic disc characteristics, stereoscopic disc photograph for three-dimensional evaluation is normally recommended. Many studies have compared the diagnostic ability of using stereoscopic versus monoscopic disc photographs to detect multiple optic disc characteristics, and they have reported varying and sometimes controversial results [13, 14]. A recent paper by the Glaucomatous Optic Neuropathy Evaluation (GONE) Project found that intraobserver and interobserver agreements for determining glaucoma, including vertical cup-to-disc ratio and RNFL defect detection, were similar between monoscopic and stereoscopic disc photographs in experienced observers [15, 16]. They then explored the factors associated with underdiagnosis of glaucoma in trainees using monoscopic disc photograph. The most common factor was inability to identify RNFL defect, followed by unseen disc hemorrhage, neural rim loss, and vertical cup-to-disc ratio [17]. These results were more pronounced in ophthalmology trainees than in fully trained ophthalmologists. In our study, two-thirds of glaucoma patients were diagnosed using C-DP by trainees, with approximately 32% of the confirmed glaucoma being missed. This reflected the limitation of identifying RNFL defect and detecting glaucoma using only C-DP.

To highlight RNFL visualization, red-free disc photograph and blue reflectance RNFL with confocal scanning laser ophthalmoscopy were assessed and compared with C-DP [18]. They found that both red-free and blue reflectance images provided better sensitivity than C-DP; however, specificity was not statistically significantly different from C-DP. Marlow et al. applied an image-processing technique to analyze glaucoma progression [19]. Baseline and subsequent optic disc photographs were autoaligned and subtracted to show differences in optic disc characteristics. For glaucoma detection, the objective of our study, this technique could also be applied. However, this technique is unable to deliver prompt glaucoma diagnosis, since it requires at least 2 photographs to detect changes. In addition, this method requires good quality images and complicated manual processes. In this study, we applied HDR concept using a wide range of luminosities to create a final image with more visible details, especially to highlight the RNFL and the characteristics of the optic disc. There are several advantages of HDR concept over original disc photograph. First, HDR does not require a good quality image, as HDR can enhance image quality by adjusting parameters. As a result, images with media opacity will benefit from this technique (Figures 3(c), 3(d), 3(g) and 3(h)). In addition, the background of fundus in high myopia and tigroid fundus usually interfere with the evaluation of RNFL. HDR can emphasize the visualization of RNFL in such cases (Figures 3(e) and 3(f)). Furthermore, HDR requires no special device other than software that is autoadjustable and normally available free of charge. Using the HDR application, the sensitivity of glaucoma detection was significantly higher than using C-DP. In this study, HDR-DP was created by adjusting the light exposure from 1 original C-DP image, because true HDR technology is difficult to obtain due to motion artifact. While not true for HDR technology, HDR-DP modification facilitates the creation of final images with broader dynamic ranges than those of C-DP images [11].

However, HDR-DP had lower specificity than C-DP, as well as average sensitivity for RNFL defect in each quadrant in the glaucoma group. This finding was possibly due to the higher false-positive rate among normal disc photographs and glaucoma suspect photographs. Both slit defect mimicking true wedge-shaped RNFL defect and unrecognized defect in diffuse RNFL loss images could be the explanation for high false-positive results. Differences between the HDR-DP and C-DP techniques for RNFL defect detection in each quadrant were not observed. This may be explained by the fact that HDR potentially improved visualization of not only the RNFL but also the disc characteristics, which may have led to the bias of RNFL defect at the corresponding disc area.

Interobserver agreement in our study was 0.33 (95% CI: 0.21 to 0.45) and 0.43 (95% CI: 0.31 to 0.56) for HDR-DP and C-DP, respectively. This was similar to Callewaert et al. that reported interobserver variability on fundus images of 0.29 for trainees and 0.37 for residents [20]. Even after training, nonexpert ophthalmologists demonstrated interobserver agreement of 0.27 [10]. Although definitions of nonexperienced clinicians/evaluators varied among studies, glaucoma experts always showed better interobserver agreement [21].

There were some limitations in our study. First, low specificity was found due to overestimation of RNFL defect in the HDR-DP group. The RNFL pattern in HDR-DP of normal and glaucoma suspect patients mimicked slit RNFL defect and may have induced false-positive results. Improvement in HDR program parameters may improve overall specificity and increase sensitivity of RNFL defect, when compared to C-DP. Secondly, we performed the study using first-year residents that had only 3 months of clinical ophthalmology training. Although we had briefly trained them on how to evaluate optic disc photograph in glaucoma, their lack of experience remained the predominant drawback. In addition, the residents may have had added difficulty with HDR interpretation, because this was the first time HDR concept was applied to disc photograph. These factors resulted in the high interobserver variations found in our study. Further study is needed using evaluators with different levels of experience. Moreover, we did not explore other specific characteristics of the optic disc that affect or may affect glaucoma detection. These optic disc characteristics should also be further investigated.

In summary, HDR-DP provided better sensitivity for glaucoma detection than C-DP, but specificity was not improved. HDR-DP might be an effective alternative glaucoma screening tool for general practitioners, nonexpert ophthalmologists, and trainees.

## Figures and Tables

**Table 1:** Demographic and clinical data of study subjects.

Demographic and clinical data	Glaucoma (*N* = 10)	Glaucoma suspect (*N* = 5)	Normal (*N* = 5)
Age (y), mean ± SD	61.0 ± 9.8	55.8 ± 12.1	47.6 ± 1.8
Female (eyes)	7	3	5
Laterality (right eye)	5	3	3
Lens status			
Clear	0	3	3
Nuclear sclerosis grade 1	7	2	2
Nuclear sclerosis grade 2	2	0	0
Pseudophakia	1	0	0
Cup-to-disc ratio, mean ± SD	0.7 ± 0.1	0.7 ± 0.1	0.3 ± 0.0
Spherical equivalent (*D*), mean ± SD	0.1 ± 0.1	2.2 ± 0.2	0.1 ± 0.7
Intraocular pressure (mmHg), mean ± SD	14.0 ± 3.2	12.2 ± 2.9	15.6 ± 3.7
Average RNFL thickness (*μ*m), mean ± SD	74.0 ± 6.1	100.2 ± 9.6	105.8 ± 17.2
Mean deviation (dB), mean ± SD	−4.1 ± 2.9	−1.5 ± 1.9	−0.4 ± 0.7
Pattern standard deviation (dB), mean ± SD	4.1 ± 2.1	2.4 ± 0.9	1.5 ± 0.3

**Table 2:** Comparison of averaged sensitivity and specificity between C-DP and HDR-DP.

	Mean ± SD (95% CI)		Mean difference ± SD (95% CI)	*p* value^∗^
	C-DP	HDR-DP		
Sensitivity (%)	68.0 ± 19.3 [54.2, 81.8]	87.0 ± 13.4 [77.4, 96.6]	19.0 ± 19.7 [4.91, 33.1]	0.014
Specificity (%)	75.0 ± 17.2 [62.7, 87.3]	46.0 ± 28.8 [25.4, 66.6]	−29.0 ± 21.8 [ 44.6, −13.4]	0.002
PPV (%)	75 .0 ± 15.1 [64.2, 85.8]	64.4 ± 15.4 [53.3, 75.4]	−10.6 ± 16.2 [−22.2, 0.9]	0.068
NPV (%)	72.1 ± 12.3 [63.3, 81.0]	79.7 ± 19.7 [65.6, 93.7]	7.5 ± 23.3 [−9.1, 24.2]	0.332

^∗^ *p* values less than 0.05 indicate statistical significance. SD: standard deviation; CI: confidence interval; C-DP: color optic disc photography; HDR-DP: high dynamic-range concept optic disc photography; PPV: positive predictive value; NPV: negative predictive value. Mean difference is the comparison of each item between HDR-DP and C-DP (HDR − C).

**Table 3:** Comparison of averaged sensitivity of RNFL defect detection between C-DP and HDR-DP in each quadrant of the glaucoma group.

Area	Method	Mean ± SD	Mean difference^∗^ ± SD (95% CI)	*p* value of mean difference
Nasal	C-DP	72.0 ± 9.2	10.0 ± 16.3 (−1.7, 21.7)	0.085
	HDR-DP	62.0 ± 14.8		
Superonasal	C-DP	58.0 ± 7.9	3.0 ± 6.7 (−1.8, 7.8)	0.193
	HDR-DP	55.0 ± 9.7		
Superotemporal	C-DP	59.0 ± 17.9	7.0 ± 18.9 (−6.5, 20.5)	0.271
	HDR-DP	52.0 ± 11.4		
Temporal	C-DP	78.0 ± 4.2	6.0 ± 11.7 (−2.4, 14.4)	0.140
	HDR-DP	72.0 ± 10.3		
Inferotemporal	C-DP	41.0 ± 16.6	−6.0 ± 22.7 (−22.2, 10.2)	0.425
	HDR-DP	47.0 ± 17.0		
Inferonasal	C-DP	57.0 ± 15.7	1.0 ± 13.7 (−8.8, 10.8)	0.823
	HDR-DP	56.0 ± 15.1		

^∗^Mean difference is the comparison of each item between HDR-DP and C-DP (HDR − C).
